# Sequential organ failure assessment score is an excellent operationalization of disease severity of adult patients with hospitalized community acquired pneumonia – results from the prospective observational PROGRESS study

**DOI:** 10.1186/s13054-019-2316-x

**Published:** 2019-04-04

**Authors:** Peter Ahnert, Petra Creutz, Katrin Horn, Fabian Schwarzenberger, Michael Kiehntopf, Hamid Hossain, Michael Bauer, Frank Martin Brunkhorst, Konrad Reinhart, Uwe Völker, Trinad Chakraborty, Martin Witzenrath, Markus Löffler, Norbert Suttorp, Markus Scholz, The PROGRESS Study Group, Stefan Angermair, Christoph Arntzen, Lorenz Balke, Robert Bals, Michael Benzke, Ayhan Berber, Frank Bloos, Martin Buchenroth, Lea Deterding, Nicolas Dickgreber, Oleg Dmitriev, Hermann Druckmiller, Holger Flick, Ulrike Föllmer, Julia Freise, Carmen Garcia, Sven Gläser, Christian Grah, Simone Hamberger, Karsten Hartung, Barabara Hauptmeier, Matthias Held, Frederik Hempel, Iris Hering, Carola Hobler, Andreas Hocke, Ursula Hoffmann, Henning Kahnert, Oliver Kanwar, Lena Kappauf, Charlotte Keller, Nils Keller, Walter Knüppel, Eva Koch, Martin Kolditz, Christine Krollmann, Cornelia Kropf-Sanchen, Josefa Lehmke, Christian Lensch, Andreas Liebrich, Achim Lies, Katrin Ludewig, Lena-Maria Makowski, Phillippr Mayer, Brigitte Mayer, Agata Mikolajewska, Anne Moeser, Thomas Müller, Michaela Niebank, Markus Niesen, Tim Oqueka, Wulf Pankow, Judith Pannier, Claus Peckelsen, Mathias Plauth, Mathias Pletz, Jan Pluta, Kalina Popkirova, Jessicar Rademache, Mirja Ramke, Felix Rosenow, Stefan Rüdiger, Bernhard Ruf, Jan Rupp, Bernhard Schaaf, Tom Schaberg, Marianne Schelle, Patrick Schmidt-Schridde, Galina Schott, Barbara Schröder, Tetyana Shchetynska-Marinova, Michael Simpfendörfer, Thomas Spinner, Norbert Suttorp, Dorina Thiemig, Daniel Thomas-Rüddel, Markus Unnewehr, Barbara Wagener, Gudrun Wakonigg, Deborah Wehde, Hubert Wirtz, Charite ICU-Teams

**Affiliations:** 1 University of Leipzig, Institute for Medical Informatics, Statistics and Epidemiology (IMISE), Härtelstr. 16-18, 04107 Leipzig, Germany; 2 Department of Infectious Disease and Respiratory Medicine, Charité – Universitätsmedizin Berlin, corporate member of Freie Universität Berlin, Humboldt-Universität zu Berlin, and Berlin Institute of Health, Campus Virchowklinikum, Augustenburgerplatz 1, 13353 Berlin, Germany; 3 Faculty of Informatics / Mathematics, HTW Dresden University of Applied Sciences, Friedrich-List-Platz 1, 01069 Dresden, Germany; 4 Jena University Hospital, Integrated Biobank Jena (IBBJ) and Institute of Clinical Chemistry and Laboratory Diagnostics, Am Klinikum 1, 07740 Jena, Germany; 5 Technische Hochschule Mittelhessen, University of Applied Sciences, Life Science Engineering, Wiesenstr. 14, 35390 Gießen, Germany; 6 Department of Anesthesiology and Intensive Care Medicine, Jena University Hospital, Am Klinikum 1, 07747 Jena, Germany; 7 Center for Clinical Studies and Department of Anesthesiology and Intensive Care Medicine, Jena University Hospital, Am Klinikum 1, 07747 Jena, Germany; 8 Jena University Hospital, Am Klinikum 1, 07747 Jena, Germany; 9 Department Functional Genomics, Interfaculty Institute of Genetics and Functional Genomics, University Medicine Greifswald, Felix-Hausdorff-Str. 8, 17475 Greifswald, Germany; 10 University Hospital Giessen, Institute for Medical Microbiology, Schubertstr. 81, 35392 Gießen, Germany; 11 Department of Infectious Disease and Respiratory Medicine, Charité – Universitätsmedizin Berlin, corporate member of Freie Universität Berlin, Humboldt-Universität zu Berlin, and Berlin Institute of Health, Charitéplatz 1, 10117 Berlin, Germany

**Keywords:** Clinical epidemiology, Biomarker, Severity score, Prospective clinical study, Infectious disease, Lung disease

## Abstract

**Background:**

CAP (Community acquired pneumonia) is frequent, with a high mortality rate and a high burden on health care systems. Development of predictive biomarkers, new therapeutic concepts, and epidemiologic research require a valid, reproducible, and quantitative measure describing CAP severity.

**Methods:**

Using time series data of 1532 patients enrolled in the PROGRESS study, we compared putative measures of CAP severity for their utility as an operationalization. Comparison was based on ability to correctly identify patients with an objectively severe state of disease (death or need for intensive care with at least one of the following: substantial respiratory support, treatment with catecholamines, or dialysis). We considered IDSA/ATS minor criteria, CRB-65, CURB-65, Halm criteria, qSOFA, PSI, SCAP, SIRS-Score, SMART-COP, and SOFA.

**Results:**

SOFA significantly outperformed other scores in correctly identifying a severe state of disease at the day of enrollment (AUC = 0.948), mainly caused by higher discriminative power at higher score values. Runners-up were the sum of IDSA/ATS minor criteria (AUC = 0.916) and SCAP (AUC = 0.868). SOFA performed similarly well on subsequent study days (all AUC > 0.9) and across age groups. In univariate and multivariate analysis, age, sex, and pack-years significantly contributed to higher SOFA values whereas antibiosis before hospitalization predicted lower SOFA.

**Conclusions:**

SOFA score can serve as an excellent operationalization of CAP severity and is proposed as endpoint for biomarker and therapeutic studies.

**Trial registration:**

clinicaltrials.gov NCT02782013, May 25, 2016, retrospectively registered.

**Electronic supplementary material:**

The online version of this article (10.1186/s13054-019-2316-x) contains supplementary material, which is available to authorized users.

## Background

CAP (Community acquired pneumonia) is one of the most frequent infectious diseases worldwide, contributing to more than a quarter million hospital admissions per year in Germany [1, 2]. Almost half of all patients with severe CAP developed sepsis, with a total mortality of about 13% [3]. Thus, there is an urgent need to develop new therapeutic strategies against CAP and for early detection of a severe disease course.

Observational studies are crucial for research on molecular pathomechanistic concepts and identification of new biomarkers, complementing efforts of experimental work in vitro and in vivo. Eventually, clinical trials are necessary to prove the effects of new therapies or the utility of new diagnostic or prognostic markers [4]. For both, observational studies and clinical trials, high quality endpoints are essential. When overall mortality is relatively low, measures of disease severity at different time points may be an important alternative [5]. Such an operationalization should be objectively assessable since clinical judgment may under- or overestimate severity of CAP [6, 7]. The operationalization should be of quantitative nature, capture relevant pathomechanistic aspects, and largely conform to clinical judgment. The goal of our analyses presented here was to identify such an operationalization for CAP severity of hospitalized patients.

Common endpoint for studies of hospitalized CAP is short-term mortality. Expansion of this endpoint by specific intensive care treatment is becoming accepted [5, 7–10]. A number of scores are described in the literature, which may serve as candidates for operationalization of CAP severity. These comprise scores initially developed to predict CAP or sepsis outcome (e.g. IDSA/ATS minor criteria [11], C(U)RB65 [12, 13], Halm [5, 14], PSI [15], SCAP [9], and SMART-COP [7]) as well as scores designed to longitudinally describe disease severity (e.g. qSOFA [16], SIRS [17], and SOFA [18]). Despite these efforts to develop clinical scoring systems, assessment of CAP severity still mainly relies on sound clinical judgment, which however is observer dependent and qualitative, therefore not suitable for quantitative analyses [19]. Thus, the current lack of a validated operationalization of CAP severity throughout the course of treatment in the hospital may hamper clinical research [20]. Analyses of well-defined and deeply characterized cohorts of CAP patients are required to improve this situation.

The ongoing PROGRESS study is conducted as a large-scale multi-centric observational study of hospitalized patients with CAP to establish a comprehensive database for high quality clinical and molecular research [21]. We use this database to evaluate the above-mentioned scoring systems regarding their potential to objectively operationalize CAP severity. The aim of this analysis is to identify suitable endpoints for subsequent biomarker research.

## Methods

### Study subjects

**Fig. 1:**
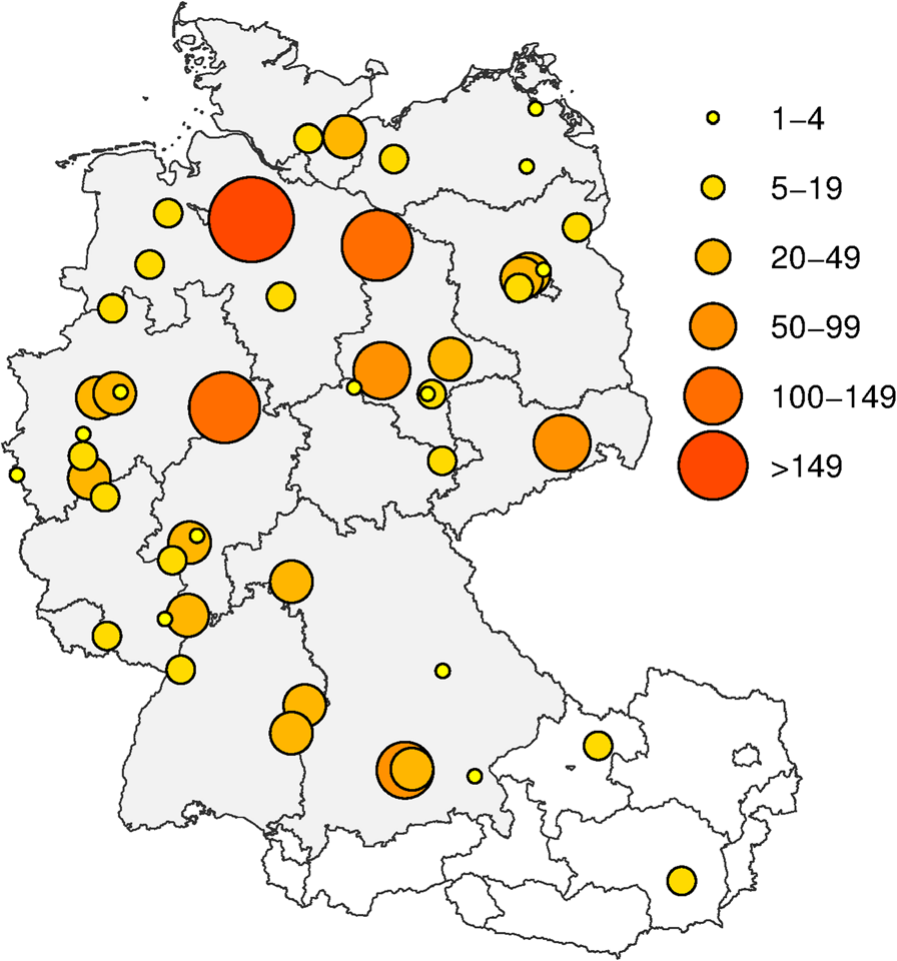
Study sites, hospitals in Germany (*N* = 54) and Austria (*N* = 2) participating in the study (Size and color of circles indicate the number of patients collected by the corresponding site)

Analyses in this manuscript were based on data of 1532 subjects recruited in the PROGRESS study (clinicaltrials.gov: NCT02782013). Details on study design and procedures can be found elsewhere [21]. In short, PROGRESS is a multicenter observational study with biomaterial asservation of CAP patients admitted to hospitals in Germany and Austria (Fig. 1). The major aim of the PROGRESS study is to identify clinical and molecular-genetic factors determining or predicting severe disease courses in a hypothesis free manner. A total sample size of 3000 is envisaged.

Here, we analyze data of PROGRESS patients from the first half of recruitment, with the aim to develop a suitable operationalization of CAP severity as endpoint for later molecular-genetic research.

Patients of the PROGRESS study were enrolled at general wards (75%), intensive care units (ICU, 13%), and emergency departments (10%). Data were collected by trained study nurses and documented using standardized web-based case report forms (eCRF). The protocol was approved by the ethics committee of the University of Jena (2403–10/08) and by locally responsible ethics committees for each study site. Requirements of the Declaration of Helsinki [22] and the ICH-GCP guideline [23] were met.

All study participants suffered from confirmed CAP and were at least 18 years of age. Written informed consent was obtained from patients or their legal representatives. CAP was defined as working diagnosis of CAP provided by the enrolling physician, pulmonary infiltrate detected by chest X-ray, and at least two of the following five symptoms: 1) fever, 2) cough, 3) purulent sputum, 4) shortness of breath or need for respiratory support, or 5) crackling or rales on auscultation, dullness to percussion, or bronchial breathing.

Hospital acquired pneumonia was avoided by exclusion of patients hospitalized due to pneumonia for more than 48 h before enrollment and patients hospitalized for any other reason within the last 28 days. PROGRESS focuses on immune competent patients, thus excluding immune-compromising comorbidities and treatments. For details on exclusion criteria and their frequencies see Additional file 1. A screening log was used to estimate the frequency of exclusion criteria and other reasons for non-participation [21]. Detection of pathogens relied on tests carried out in routine care: blood culture, culture of respiratory materials, antigen tests for *S. pneumoniae* and *L. pneumophila*, and Influenza rapid tests. Results were documented as positive if so considered by the treating physician.

### Study-specific assessments

Patient baseline data comprised socio-demographic, anthropometric, and anamnestic information. Risk factors for pneumonia such as smoking history, previous antibiotic use, tube feeding, and mild or newly administered immunosuppression not counting as exclusion criteria were recorded [24, 25].

**Fig. 2:**
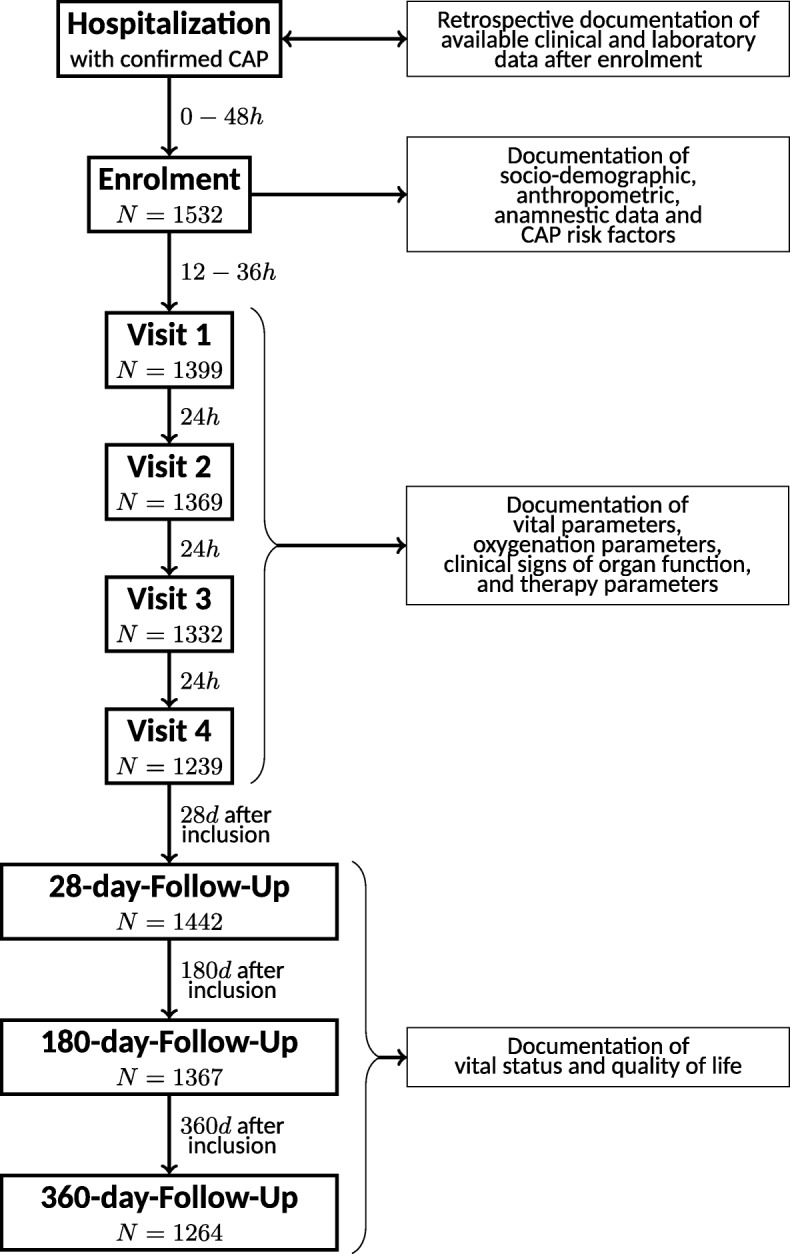
Flow chart of study procedures

Initial assessment at enrollment (d0) was followed by four study visits (d1 through d4). On d0 through d4, vital parameters (e.g. heart & respiratory rate, body temperature), oxygenation parameters (e.g. blood gas analysis, pulse oximetry), markers for organ function (e.g. bilirubin, creatinine, Glasgow coma scale, use of catecholamines), and other relevant parameters (e.g. detected pathogens, antibiotic treatment, ventilation, treatment on ICU) were assessed and documented and blood samples were taken. Documentation was completed by follow-up at days 28, 180, and 360 post enrollment. A flow chart of our study is shown in Fig. 2. Exclusion criteria and their frequencies can be found in Additional file 1.

### Primary endpoint

As mortality in the PROGRESS cohort is low, a composite primary endpoint (PE) was used for comparison of score’s ability to operationalize CAP severity. PE was defined as death within 28 days by any cause, or transfer to ICU. To ensure that transfer to ICU was not solely due to considerations other than deterioration of CAP, we required new CAP-specific treatment in addition to ICU transfer: Substantial respiratory support (ventilation, extracorporeal oxygenation, oxygen supplementation ≥6 l per minute, except for patients with home ventilation), treatment with catecholamines (any dose of adrenalin, epinephrine, noradrenalin, norepinephrine, dopamine, or dobutamine), or dialysis (except for patients with chronic kidney disease). Similar concepts have been proposed before [5, 7–10, 26].

### Candidate scores for operationalization of CAP severity

The literature was reviewed for established pneumonia and sepsis related outcome and severity scores with potential for operationalization of CAP severity. Of these, we selected scores for which data required for calculation are available in PROGRESS at least at the day of enrolment. If comparison of highly similar scores was available in the literature, only the superior score was selected. Scores defined for clearly different settings or which were too similar to our primary endpoint were excluded.

### Statistical analysis

#### Association of severity scores and primary endpoint

Performance of selected scores in describing current CAP severity was assessed by receiver operating characteristics (ROC) for detecting presence (cases) vs. absence (controls) of PE at d0. Area under ROC curves, Youden-statistics (sensitivity+specificity-1), and corresponding confidence intervals were calculated. Differences between areas under ROC curves for different scores were tested for significance. Inspired by Fine et al., missing parameters were replaced by uncritical values to accommodate score calculation [27]. For time series analyses of SOFA and PE, missing parameters were imputed by “last observation carried forward”.

Prediction performance of scores is compared by calculating net reclassification improvements (NRI) of cases and controls separately [28]. The NRI is the difference of relative prediction improvement and prediction deterioration when comparing two prediction models. As an example, NRI = 0.1 implies that the difference of patients better classified by model 1 and patients better classified by model 2 is 10%, i.e. the statistics measures the net improvement of prediction.

#### Comparison with other cohorts

To assess the generalizability of our results, we compare our PROGRESS cohort with the general population of hospitalized CAP patients in Germany based on the 2014 AQUA report and with the GenIMS study which is similar to our study [2, 29]. Comparisons were performed using standard descriptive methods and tests.

#### Analysis of pre-clinical risk factors

We analyzed the impact of known risk factors of disease severity and progression on SOFA score at enrollment, namely age, sex, BMI, smoking status, pack-years, and antibiotic treatment in the five days before hospitalization [7, 24, 25, 30, 31]. We performed uni- and multivariate linear regression analyses and tested for interaction.

All calculations were performed using the statistical software package R [32].

## Results

### Cohort description

#### Baseline characteristics and comparison with other cohorts

Of the 1532 subjects from the PROGRESS study considered here, 902 (59%) were male. Mean age of all subjects was 59 years. Further patient characteristics are shown in Table 2, comprising baseline risk factors (e.g. nursery home residency, smoking history), chronic comorbidities, clinical state at enrollment, treatment, and outcome. For 994 patients (65%) results of microbiological analysis were available, with 103 testing positive for Streptococcus pneumoniae (10.4% of those tested).

We compared our PROGRESS study with the similar GenIMS study [29] and the overall population of hospitalized CAP patients in Germany described in the annual AQUA report [2]. Percentage of males was roughly similar in all three samples but PROGRESS patients were younger. Risk factors like nursing home residency and chronic bed confinement were less common in PROGRESS. Comparison of clinical characteristics is shown in Table 2.

#### Primary endpoint

**Fig. 3:**
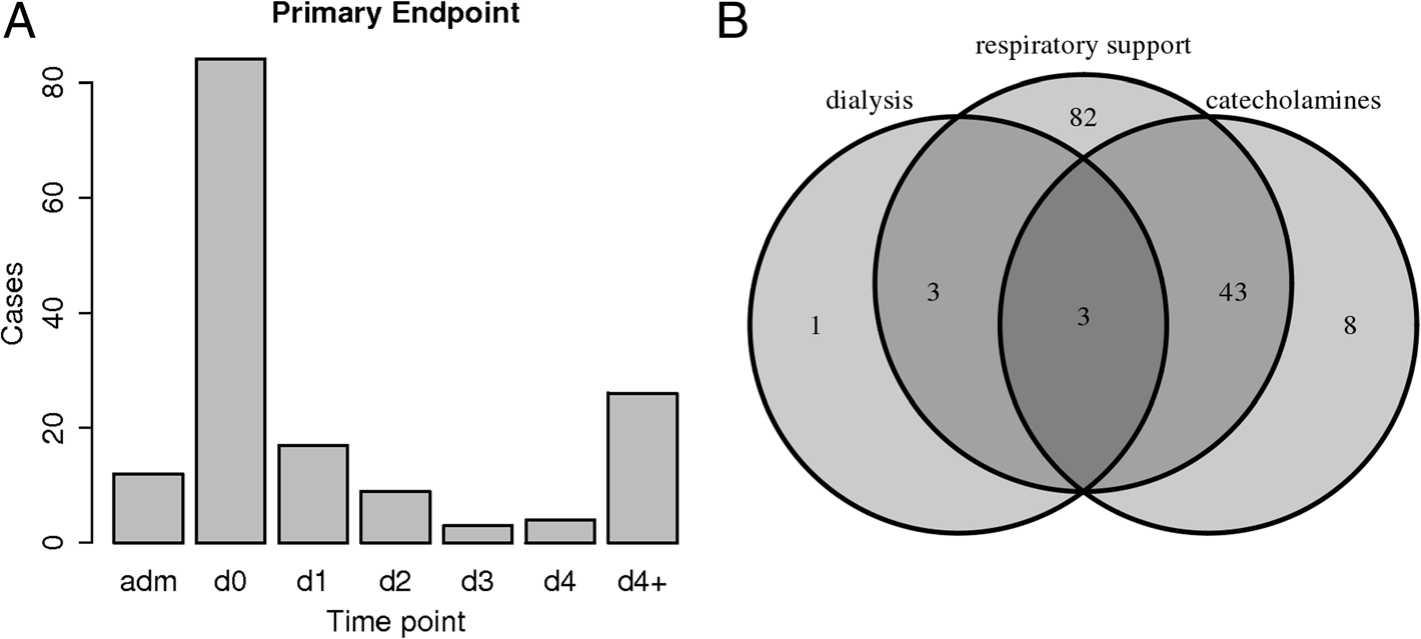
Primary Endpoint (PE) in the PROGRESS study. **a** Distribution of observed PE states across study events (adm = admission to hospital, d0 = enrollment, d1 = study visit 1, d2 = study visit 2, d3 = study visit 3, d4 = study visit 4, d4+ = time between study visit 4 and 28d follow-up. **b** Frequencies of specific treatments qualifying for the PE

28-day mortality was low in our cohort (*N* = 35, 2.3%). Therefore, most PE cases were caused by “qualified ICU”, i.e. stay on ICU with at least one of the following treatments: Ventilation (*N* = 112), catecholamines (*N* = 54), oxygen supplementation with at least 6 l/min (*N* = 51), extracorporeal oxygenation (*N* = 4), or dialysis (*N* = 7). More than one criterion may be fulfilled concurrently. In total *N* = 155 (10.1%) patients suffered the endpoint. See Fig. 3 for overlap of treatments and for distribution of PE occurrence over time.

#### Candidate severity scores

Review of the literature led to selection of the following scores as candidates for operationalization of CAP severity: IDSA/ATS minor criteria [11], CRB-65 [12], CURB-65 [13], Halm [5, 14], PSI [15], qSOFA [16], SIRS [17], SCAP [9], SMART-COP [7], and SOFA [18]. An overview including considered deterioration of organ functions is provided in Table 1.

**Table 1:** Overview of scoring systems considered for operationalization of CAP severity

Score	System								
	C(U)RB -65	Halm	IDSA/ATS minor criteria	PSI	qSOFA	SCAP	SIRS	SMART-COP	SOFA
Lung	✓	✓	✓	✓	✓	✓	✓	✓	✓
Cardio-vascular system	✓	✓	✓	✓	✓	✓	✓	✓	✓
Central nervous system	✓	✓	✓	✓	✓	✓	✓	✓	✓
Kidney	(✓)	–	✓	✓	–	✓	✓	–	✓
Liver	–	–	–	✓	–	✓	–	✓	✓
Coagulation	–	–	✓	–	–	–	✓	–	✓
Metabolism	–	–	–	–	–	–	✓	✓	–
Status of Infection	–	✓	✓	✓	–	–	✓	–	–
General state of health	✓	–	–	✓	–	✓	–	–	–
# Organs/Systems assessed	4 (5)	4	6	7	3	6	7	5	6
# Categories	5 (6)	8	Quasi Cont.	Quasi Cont.	4	Quasi Cont.	4	Quasi Cont.	Quasi Cont.

Considered scoring systems differ in number of extra-pulmonary organs evaluated and number of resulting categories. Scores with 10 or more ordered categories were considered quasi-continuous (Quasi Cont.), indicating that analyses as for continuous parameters are appropriate. CRB-65 and CURB-65 are combined here but analyzed separately

All selected scores assess cardiopulmonary function and the central nervous system, but differ in their original purpose, coverage of other organ systems, and specific parameters used. For PSI, CRB-65, and CURB-65 lower score values were more prevalent in our study (see in Additional file 1: Figures S2 & S3). For SIRS, about 80% of PROGRESS patients were assigned to the second class (sepsis) while the first and third classes were allocated in a ratio of about 2:1. Descriptive statistics for IDSA/ATS minor criteria, Halm, SCAP, SMART-COP, and SOFA are also provided in Table 2. Again, most patients show lower score values. The distribution of SOFA scores at enrollment (and at subsequent study visits) is shown in Additional file 1: Figure S4, with the highest frequency for two SOFA points.

**Table 2:** Comparison of Patients in PROGRESS with Patients in AQUA and GenIMS

		PROGRESS	AQUA	GenIMS
	N, number of cases	1532	258,049	1886
	Age, mean (sd)	59 (18.3)	73 (*p* = 6.9 × 10^− 151^)^1^	68 (*p* = 5.1 × 10^−67^) ^1^
	Age > =60 in % (N)	55% (836)	82% (*p* = 2.1 × 10^− 177^)^2^	NA
	Male sex	59% (902)	57% (*p* = 0.13)^2^	52% (*p* = 9 × 10^−5^)^2^
Comorbidities and other risk factors	Nursery home resident	1.5% (23)	20.7% (*p* = 8.5 × 10^−76^)^2^	6.2% (*p* = 1.7 × 10^−11^)^2^
	Smoking history	66% (942)	NA	66% (*p* = 0.84)^2^
	Antibiotic treatment before admission to hospital, missings excluded	419 (28%)	NA	NA
	Antibiotic treatment during the last 5 days, missings excluded	224 (15%)	NA	NA
	Antibiosis before admission (in days), mean (sd) median	0.77 (2.4) 0	NA	0.8 (1.4) 0 (*p* = 0.6169)^1^
	Chron. Lung disease	30% (458)	NA	26% (*p* = 0.014)^2^
	Chron. cardiovascular disease	26% (399)	NA	26% (*p* = 0.70)^2^
	Chron. renal disease	10% (153)	NA	NA
	Chron. liver disease	2.2% (33)	NA	NA
	Chron. cerebrovascular disease	5.7% (87)	NA	NA
	Tumor disease	8.8% (134)	NA	NA
	Any of these comorbidities	52% (789)	NA	NA
	Chron. bed confinement	0.9% (14)	21.4% (*p* = 2.5 × 10^−84^)^2^	NA
Condition/ treatment at enrollment	Disoriented*	5.8% (88)	32.7% (*p* = 4.7 × 10^−110^)^2^	NA
	Low blood pressure*, (Systolic < 90 mmHg or Diastolic < =60 mmHg)	19% (288)	NA	NA
	High respiratory rate*, (> = 30 1/min)	6.6% (62)	NA	NA
	Vasopressors*, (> = 30 1/min)	2.9% (44)	NA	NA
ICU and ventilation	Mechanical ventilation within 28d, missings treated as ‘No’	9.5% (145)	9.2% (*p* = 0.1618)^2^	7.3% (*p* = 0.024)^2^
	Mechanical ventilation within 28d, missings excluded	17.4% (145)	9.2% (*p* = 2.8 × 10^−20^)^2^	7.3% (*p* = 2.2 × 10^− 15^)^2^
	ICU within 28d, missings treated as ‘No’	17% (259)	NA	16% (*p* = 0.51)^2^
	ICU within 28d, missings excluded	24% (259)	NA	16% (*p* = 1.2 × 10^−07^)^2^
Scores and Endpoints	Length of hospital stay (LOHS) in (d): mean (sd) median	10.0 (15.5) 8	NA	7.3 (5.0) 6 (*p* = 9.7 × 10^−12^) ^1^
	LOHS (1–7/8–14/15–21/> 21) in %	46.3/40.7/7.8/5.2	42.5/38.6/10.7/6.8 (*p* = 1 × 10^−04^)^3^	NA
	Primary endpoint	10.1% (155)	NA	NA
	In-hospital mortality	2.3% (33)	13.0% (*p* = 1.3 × 10^−15^)^2^	NA
	28-day mortality	2.3% (35)	NA	NA
	90-day mortality	3.5% (54)	NA	11.5% (*p* = 2.4 × 10^−17^)^2^
	CRB-65* (0–4) in %	41.3/40.3/14.8/3.4/0.1	17.2/52.5/23.1/4.5/2.7 (*p* = 2.1 × 10^−88^)^3^	NA
	CURB-65* (0–5) in %	37.0/30.0/21.2/9.2/2.5/0.1	NA	NA
	Halm* mean, (sd)	1.8 (1.1)	NA	NA
	IDSA/ATS* mean, (sd)	2.0 (1.4)	NA	NA
	PSI* (1–5) in %	23.4/22.9/20.9/23.9/9.0	NA	30.7/25.2/33.4/10.8 (*p* = 1.9 × 10^−17^)^3^
	qSOFA* (0–3) in %	44.8/44.8/9.8/0.7	NA	NA
	SCAP* mean, (sd)	7.8 (7.5)	NA	NA
	SIRS* (1/2/3) in %	13.2/80.2/6.7	NA	NA
	SMART-COP* mean, (sd)	2.1 (1.6)	NA	NA
	SOFA* mean, (sd)	2.9 (2.2)	NA	NA

Characteristics of the PROGRESS patient population compared to the general population of hospitalized CAP patients in Germany (AQUA) and another large observational study of CAP patients (GenIMS)

*NA* data not available

* = value at the time of enrollment; ** = classes I and II are pooled together

^1^ = p-value of a one sample t-test; ^2^ = p-value of a chi-square test; ^3^ = p-value of a Mann-Whitney U-test

### Scores for operationalization of CAP severity

#### Score comparison at enrollment

To evaluate their potential for operationalization of CAP severity, we compared ten established scores for their ability to identify PE at d0.

**Fig. 4:**
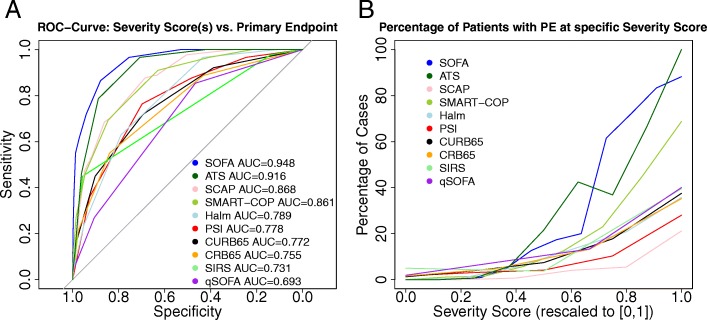
Performance of scores regarding PE prediction. **a** Receiver operating characteristics for severity scores at enrollment. **b** Percentage of patients with PE in dependence on severity scores: Severity scores were rescaled to the unit interval for this purpose. For SOFA we pooled scores > 10, for Halm > 5 and for SMART-COP > 8 in order to deal with sparsely filled score classes. For SCAP, quintiles were used

Figure 4a displays corresponding ROC curves, showing a clearly superior diagnostic value of the SOFA score, indicated by largest AUC and maximum Youden index (for further details see Table 3). All comparisons of SOFA with the other scores were statistically significant. Second and third highest AUC values were achieved by IDSA/ATS minor criteria and SCAP, respectively. Regarding NRI, SOFA is superior in classification of both, cases (with PE) and controls (without PE). For instance, for SOFA compared to second placed IDSA/ATS minor criteria, NRI for cases is 0.28 and for controls 0.17 (see Additional file 1: Table S1 for comparison of all scores versus the null model (guessing) and for the comparison of SOFA with the alternative scores)

**Table 3:** Diagnostic power of scores regarding the primary endpoint

	AUC	Sensitivity	Specificity	max Youden-Index	Cut-off	*P*-value*
SOFA	**0.95** [0.93,0.97]	0.86 [0.82,0.99]	0.88 [0.74,0.90]	**0.74**	4.5	–
IDSA/ATSmc	0.92 [0.89,0.94]	0.79 [0.78,1.00]	0.89 [0.69,0.90]	0.68	3.5	3.8 × 10^−3^
SCAP	0.87 [0.84,0.90]	**0.88** [0.66,0.94]	0.69 [0.66,0.88]	0.56	10.5	2.4 × 10^−7^
SMART-COP	0.86 [0.82,0.90]	0.70 [0.66,0.96]	0.84 [0.61,0.86]	0.54	3.5	9.2 × 10^−7^
Halm	0.79 [0.75,0.83]	0.63 [0.60,0.99]	0.79 [0.42,0.81]	0.42	2.5	3.6 × 10^−13^
PSI	0.78 [0.73,0.82]	0.76 [0.67,0.85]	0.70 [0.67,0.72]	0.46	3.5	1.4 × 10^− 14^
CURB-65	0.77 [0.72,0.82]	0.72 [0.47,0.82]	0.69 [0.67,0.91]	0.41	1.5	2.2 × 10^−14^
CRB-65	0.76 [0.70,0.81]	0.55 [0.47,0.92]	0.84 [0.43,0.86]	0.39	1.5	9.5 × 10^−15^
SIRS	0.73 [0.68,0.78]	0.45 [0.35,0.55]	**0.96** [0.95,0.97]	0.41	2.5	2.0 × 10^−19^
qSOFA	0.69 [0.64,0.74]	0.85 [0.78,0.92]	0.47 [0.44,0.49]	0.32	0.5	1.3 × 10^−21^

Analysis was based on values at enrollment in PROGRESS (d0). Scores were ordered according to their AUC. In brackets, 95%-confidence intervals are shown. Sensitivity, specificity, and Youden-index correspond to the point of the ROC curve with maximum Youden-index (cut-off), highest values are boldfaced. P-values correspond to comparisons of AUCs with that for SOFA. All alternatives show significantly inferior diagnostic value

At the upper end of score values, only SOFA and IDSA/ATS minor criteria (> 80%), followed by SMART-COP (> 60%), accumulated a larger fraction of PE patients, compared to less than 40% for the other scores (Fig. 4b). At lower values, all scores showed similar proportions of PE patients.

#### Performance of SOFA score during course of disease and dependence on risk factors

**Fig. 5:**
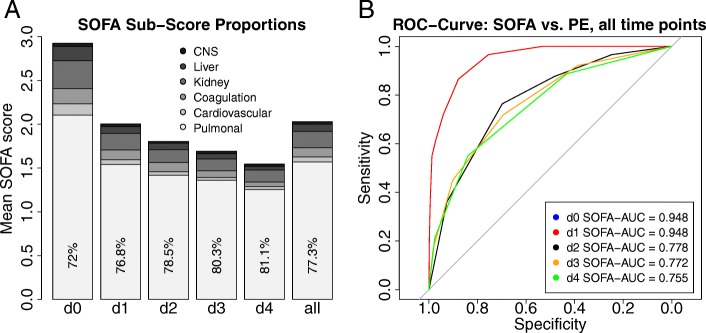
Time series data of SOFA. **a** ROC analysis of SOFA for different study time points: Diagnostic power is similar for all time points; **b** Contribution of SOFA sub-scores for day of enrollment and study visits. As expected, the pulmonary SOFA sub-score has the largest impact, which even increases during the course of therapy. Displayed numbers refer to the percentage of the pulmonary SOFA sub-score

To evaluate the value of the SOFA score for operationalization of CAP severity at different time points, longitudinal data of the PROGRESS study were considered. ROC analyses performed separately for time of enrollment (d0) and each study visit (d1 through d4) showed uniformly high diagnostic power (Fig. 5a). All ROC curves achieved AUC values above 90%. The contribution of SOFA sub-scores to the overall SOFA score is shown in Fig. 5b for all time points. As expected due to the large fraction of patients with less severe CAP, the pulmonary SOFA sub-score contributed most. The proportion was somewhat lower for the time of enrollment and increases thereafter. Overall, SOFA decreases with time and extrapulmonary sub-scores improved prior to the pulmonary sub-score. The second largest contributor to the SOFA score was the kidney sub-score with a mean contribution of 9.2%.

We analyzed the discriminative power of the SOFA in dependence on age and comorbidities. Regarding age, we observed a uniform performance across age groups (AUC: 0.94 (< 40 years), 0.97 (40–59 years), 0.93 (60–79 years), 0.95 (≥80 years). Performance is only slightly reduced in patients with comorbidities (AUC = 0.97 without comorbidities, AUC = 0.93 with comorbidities).

### Risk factors influencing SOFA score at admission

Since the SOFA score emerged as a strong candidate for operationalization of CAP severity, we analyzed known CAP risk factors regarding their influence on the score at enrollment. Univariate regression models showed significance for age (beta = 0.034, *p* = 2.0 × 10^− 30^), sex (beta = − 0.77, *p* = 5.6 × 10^− 12^, higher SOFA in males), pack-years (beta = 0.020, *p* = 4.6 × 10^− 11^), and antibiotic treatment prior to hospitalization (beta = − 0.94, *p* = 2.7 × 10^− 14^). BMI (beta = 0.017, *p* = 0.058) and current smoking (beta = − 0.17, *p* = 0.17) were not significant.

All four univariate significant factors remained significant in multivariate analysis (age: beta = 0.029, *p* = 4.8 × 10^− 23^, sex: beta = − 0.50, *p* = 2.5 × 10^− 6^, pack-years: beta = 0.0085, *p* = 4.2 × 10^− 3^, antibiotic treatment prior to hospitalization: beta = − 0.51, *p* = 1.2 × 10^− 5^). None of the possible bivariate interaction terms were significant, indicating independent and additive effects. The multivariate model explains 14% of the total variance of SOFA. As expected, high age, male sex, and pack-years were predictors for more severe disease. Patients with antibiotic treatment prior to hospitalization tended to have lower initial SOFA scores.

## Discussion

Operationalizing severity of community-acquired pneumonia is important for risk management and biomarker development. It is a major goal of our study to analyze, which of several scores best represents clinical decision-making and can serve as operationalization of CAP severity. Therefore, we compared clinical scores established to describe disease severity or predict outcome in pneumonia or sepsis. SOFA outperformed all competing scores and showed uniform diagnostic power during the course of CAP. In agreement with the literature, we identified age, sex, pack-years, and antibiotic treatment prior to hospital admission as major factors influencing initial SOFA scores [7, 24, 25, 30, 31].

Our strategy to develop a good operationalization of disease severity was to compare the discriminative power of clinical scores with respect to an *objectively severe disease state* defined by 28-day mortality or qualified intensive care treatment [5, 7–10]. We used such a combined endpoint in our study since mortality was low. The combined endpoint is very likely to be better predictable than mortality alone since available scores typically use parameters assessed by clinicians to decide on necessity of intensive care, while mortality may depend on circumstances not completely covered. This could explain somewhat lower prediction performance reported in the literature [33].

A literature search revealed a variety of possible candidate scores for operationalizing CAP severity (see Table 1). Although all of them are related to infectious diseases, there are some differences with respect to their original clinical purpose: CURB-65, CRB-65, and PSI were designed to predict mortality of CAP [12, 13, 15]. SIRS was developed to generally distinguish infection, sepsis, and severe sepsis, so that one can expect that it has discriminative power to distinguish mild from severe disease courses in CAP [17]. IDSA/ATS criteria were developed to identify patients requiring treatment on ICU. In contrast, the SOFA score is not originally defined to predict outcome, but to describe the sequence of complications in distinct organs [18]. However, it is considered to be useful for the prediction of outcome in critically ill patients [34]. The qSOFA is proposed as a simplified version of SOFA and was introduced into the new sepsis-3 definition [16]. Halm score is proposed as a summary of criteria of clinical stability. It outperformed CURB-65, IDSA/ATS stability criteria, and CRP regarding prediction of mortality of CAP patients and other severe disease outcomes [5, 14]. SMART-COP is proposed as an improvement of PSI and CURB-65 to predict requirement of intensive care for pneumonia patients [7]. Finally, SCAP was developed to predict critical time courses of pneumonia [9]. A Cochrane meta-analysis shows that IDSA/ATS minor criteria, SCAP, and SMART-COP are superior to PSI and CURB-65 in predicting ICU admission and intensive care treatment of CAP patients (SOFA was not considered) [35].

Several scores were not considered in our present analysis: Expanded-CURB shows superiority in predicting 30-day mortality compared to PSI, SMART-COP, and A-DROP [36]. However, the score requires LDH and albumin, which were not available for most of our patients. A-DROP was not considered in our study due to high similarity with CURB-65 [24]. MEWS and NEWS are shown to be superior compared to qSOFA in predicting adverse outcomes but are intended as emergency scores not fitting our study population [37]. IDSA/ATS major criteria were not considered since they consist of ventilation status and septic shock, very close to our compound endpoint. Instead, we considered the IDSA/ATS minor criteria [11] which were recently proposed to predict need for treatment on ICU [8].

Discriminative power of the scores was assessed by ROC analysis and NRI regarding the presence of our primary endpoint, PE. It revealed that the SOFA score clearly outperformed all considered alternatives. IDSA/ATS minor criteria were only somewhat inferior –probably due to their similarity with SOFA but lesser quantitativeness. Alternatives following with larger distance were SCAP and SMART-COP, followed by Halm, PSI, and CURB-65. SIRS and qSOFA performed worst. Although we focused on cross-sectional analysis, our results were in line with the literature mostly studying future events: A similar performance of CURB-65, CRB-65, and PSI regarding 30-day mortality was observed by several authors in different contexts [7, 36, 38, 39] and in a meta-analysis [35]. We observed similar AUCs for our PE as observed in these studies. Comparable performances of SIRS and qSOFA were found in a population of infected patients regarding an endpoint similar to our PE [37]. Reduced performance of qSOFA compared to SOFA has been shown [33, 40]. Improvement of CRB-65 by adding oxygenation was observed by Kolditz et al. for another German CAP population [8]. Thus, a general trend appears to emerge that SOFA-like scores, covering the current state of the lung and extrapulmonary organs prone to deterioration during CAP, such as IDSA/ATS minor criteria, SMART-COP, SCAP, or Halm perform better in identifying a severe state of CAP (for an overview of organs covered by each score see Table 1). The major advantage of SOFA in our analysis was that it had superior discriminative power in the upper range of the score where the alternatives do not achieve sufficiently high positive predictive values, with IDSA/ATS minor criteria and SMART-COP as exceptions (see Fig. 4b). Of note, the SOFA score also showed discriminative power for low values, comparable to the alternatives. SOFA showed a uniform discriminative power across study days, age groups, and patients with or without comorbidities. Using SOFA, a higher percentage of patients was better classified regarding PE in comparison to the other scores as assessed by the NRI (see Additional file 1: Table S1).

Our study has some limitations. All analyses were performed on data from the PROGRESS study, a multi-center, prospective, longitudinal observational study of hospitalized CAP patients [21] which was designed for identification of potential biomarkers by analysis of several omics layers, including genetic, transcriptomic, and proteomic features, while minimizing potential confounders. Thus, the study population was younger and less comorbid in comparison to the general population of hospitalized CAP-patients in Germany [2]. Lower mortality was observed in PROGRESS, which cannot be explained by the younger age alone (analysis not shown). For our analyses presented here on operationalization of CAP severity, this limits generalizability of results to some extent and requires replication of our results in representative patient cohorts. Potentially, this patient selection may favor prediction of PE rather than mortality. However, our results were independent of patient’s age and only slightly influenced by comorbidities.

## Conclusion

The performance of the SOFA score in operationalizing CAP severity might not come as a complete surprise. Parameters corresponding to its elements are commonly assessed by clinicians to decide on status and further treatment of patients. However, as pointed out in the literature, there is no formalization of this process so far. Based on the analysis of data from the PROGRESS study, we recommend the SOFA score to assess disease severity of hospitalized CAP patients. It has the potential to greatly facilitate clinical studies due to its (semi)quantitative nature. In particular, we consider the SOFA score a suitable endpoint for biomarker research questions as addressed in the PROGRESS study. However, we must acknowledge that applicability of our findings to the general CAP population remains to be shown in a less selected patient cohort.

## Additional file

Additional file 1: Exclusion Criteria For screened patients fulfilling inclusion criteria but not enrolled in the study, exclusion criteria and their frequencies were documented. Candidate Scores for Operationalization of CAP Severity Additional details on scores considered here as candidates for operationalization of CAP severity. **Figure S1**. Age distribution in the PROGRESS cohort in comparison with AQUA. PROGRESS patients are younger than the overall CAP population described in the AQUA report. **Figure S2**. Distribution of CRB-65 on d0 in comparison with AQUA. PROGRESS patients appear to have less severe disease at enrollment than the overall CAP population described in AQUA. Lower age is partly responsible for this effect. **Figure S3**. Distribution of PSI on d0 in comparison with GenIMS. There appears to be a larger fraction of patients with less severe disease and lower mortality risk in PROGRESS compared to patients in GenIMS. **Figure S4**. Distribution of SOFA scores at different time points (d0 = enrollment, d1 = study visit 1, d2 = study visit 2, d3 = study visit 3, d4 = study visit 4). SOFA scores 7 to 24 were pooled. According to study protocol, patients with initially high disease severity were not subjected to study visits. Therefore, at visit d0 a clear shift towards higher scores was observed. Overall, there appears to be a general trend towards improved SOFA scores over time. However, a few patients still have increased SOFA values at later time points. **Table S1**. We present net reclassification improvement (NRI) for cases and controls induced by the scores compared with the null model (guessing) and corresponding NRIs of SOFA compared to the other scores. SOFA is superior to all other scores for both, cases and controls. (DOCX 156 kb)

## Abbreviations

A-DROP: Age, Dehydration, Respiratory failure, Orientation disturbance, systolic blood Pressure
AQUA: Institut für angewandte Qualitätsförderung und Forschung im Gesundheitswesen
ATS: American Thoracic Society
AUC: Area under the curve
BMI: Body Mass Index
CAP: Community Acquired Pneumonia
CRB-65: Confusion, Respiratory rate, Blood pressure, age ≥ 65 years
CRP: C-reactive protein
CURB-65: Confusion, Urea, Respiratory rate, Blood pressure, age ≥ 65 years
d0, d1, d2, d3, d4: PROGRESS study day 0 (day of enrollment) and days 1 through 4 (study visits)
eCRF: Electronic case report form
GenIMS: Genetic and Inflammatory Markers of Sepsis study
ICH-GCP: International Council for Harmonization of Technical Requirements for Pharmaceuticals for Human Use Good Clinical Practice
ICU: Intensive Care Unit
IDSA: Infectious Diseases Society of America
IDSA/ATSmc: IDSA/ATS minor criteria
MEWS: Modified Early Warning Score
NEWS: National Early Warning Score
NRI: Net Reclassification Improvement
PE: Primary Endpoint
PROGRESS: Prospective observational study on hospitalized community acquired pneumonia
PSI: Pneumonia Severity Index, also known as Fine-Score
qSOFA: quick SOFA (Sepsis-related Organ Failure Assessment)
ROC: Receiver Operating Characteristic
SCAP: Severe CAP
SIRS: Systemic Inflammatory Response Syndrome
SMART-COP: Systolic blood pressure, Multilobar chest radiography involvement, low Albumin level, high Respiratory rate, Tachycardia, Confusion, poor Oxygenation, and low arterial pH
SOFA: Sequential Organ Failure Assessment Score
