# Supplementary material for: Sequential organ failure assessment score is an excellent operationalization of disease severity of adult patients with hospitalized community acquired pneumonia – results from the prospective observational PROGRESS study
Source: Crit Care. 2019 Apr 4;23:110. doi: 10.1186/s13054-019-2316-x (PMC6450002; doi:10.1186/s13054-019-2316-x)
Supplement: Supplementary file 1 — Exclusion Criteria For screened patients fulfilling inclusion criteria but not enrolled in the study, exclusion criteria and their frequencies were documented. Candidate Scores for Operationalization of CAP Severity Additional details on scores considered here as candidates for operationalization of CAP severity. Figure S1. Age distribution in the PROGRESS cohort in comparison with AQUA. PROGRESS patients are younger than the overall CAP population described in the AQUA report. Figure S2. Distribution of CRB-65 on d0 in comparison with AQUA. PROGRESS patients appear to have less severe disease at enrollment than the overall CAP population described in AQUA. Lower age is partly responsible for this effect. Figure S3. Distribution of PSI on d0 in comparison with GenIMS. There appears to be a larger fraction of patients with less severe disease and lower mortality risk in PROGRESS compared to patients in GenIMS. Figure S4. Distribution of SOFA scores at different time points (d0 = enrollment, d1 = study visit 1, d2 = study visit 2, d3 = study visit 3, d4 = study visit 4). SOFA scores 7 to 24 were pooled. According to study protocol, patients with initially high disease severity were not subjected to study visits. Therefore, at visit d0 a clear shift towards higher scores was observed. Overall, there appears to be a general trend towards improved SOFA scores over time. However, a few patients still have increased SOFA values at later time points. Table S1. We present net reclassification improvement (NRI) for cases and controls induced by the scores compared with the null model (guessing) and corresponding NRIs of SOFA compared to the other scores. SOFA is superior to all other scores for both, cases and controls. (DOCX 156 kb) [file 13054_2019_2316_MOESM1_ESM.docx]

# Additional file 1

## Exclusion criteria:

Frequency of exclusion criteria on the basis of 2676 screened and not enrolled patients:

- No written consent of patient, 35.69%
- Hospitalization more than 48h ago, 22.68%
- Tumor disease with therapy within the last six months, 6.54%
- Massive aspiration, 6.32%
- Insufficient capacity in the clinical procedure, 5.46%
- HIV/AIDS, 5.12%
- Steroid therapy (at least 20mg Prednisolone equivalent per day since more than 14 days before inclusion), 2.95%
- Heart insufficiency NYHA-IV, 2.88%
- Non-sterodial immunosuppressive therapy within the last six months, 2.02%
- Cytostatic therapy within the last six months, 1.83%
- Acute pulmonary embolism, 1.76%
- Poststenotic pneumonia with bronchial carcinoma, 1.46%
- Therapy restriction, 0.97%
- Sepsis with extra-pulmonic focus, 0.78%
- Home mechanical ventilation over a tracheostoma, 0.75%
- Pregnancy or breastfeeding, 0.67%
- Liver insufficiency Child-C, 0.45%
- Participation in the same study at an earlier date, 0.41%
- Active tuberculosis, 0.37%
- Extra-pulmonary caused ALI/ARDS, 0.37%
- Radiotherapy within the last 6 months, 0.30%
- Bone marrow transplantation, 0.15%
- Cystic fibrosis, 0.07%

## Candidate Scores for Operationalization of CAP Severity

### CURB-65 and CRB-65

The six-state score CURB-65 was introduced to stratify CAP patients according to their risk of 30 day mortality.[1] The score consists of the clinical items confusion, urea, respiratory function, blood pressure, and age (65 years). The CRB-65 was introduced as a variant not utilizing the measurement of urea. Both, CURB-65 and CRB-65, are recommended for use as decision rules regarding inpatient or outpatient treatment.[2] CURB-65 and CRB-65 can take six and five different values, respectively.

### Halm criteria

Halm et al. presented a number of criteria of clinical stability.[3] On this basis, Akram et al. proposed a score outperforming CURB-65, ATS criteria, and CRP regarding prediction of mortality of CAP patients and other severe disease outcomes.[3, 4] The score additionally comprises status of infection measured by body temperature, resulting in eight possible categories.

### IDSA/ATS minor criteria

The Infectious Disease Society of America and the American Throacic Society published consensus guidelines on the management of community-acquired pneumonia in adults.[5] These guidelines contain criteria to support decision on admission to ICU. The major criteria are septic shock or need for vasopressors. Nine minor criteria cover relevant organ systems (lung, central nervous system, kidney, coagulation, cardiovascular system) and signs of infection in a binary fashion, leading to a quasi continuous score with ten possible values. Patients with a score ≥3 should be admitted to ICU. IDSA/ATS minor criteria are predictors of early deterioration[6] and in a before-and-after study have been shown to aid in pre-intensive care unit resuscitation in severe CAP.

### Pneumonia severity index (PSI)

PSI is constructed as a predictor of mortality for adult hospitalized CAP patients.[7–9] It has been shown that its prediction of mortality also works well for patients on intensive care units.[10] The PSI score utilizes 20 clinical parameters including age, sex, certain comorbidities, heart rate, respiratory rate, and several laboratory findings. Although being introduced as a predictor of mortality, PSI is also used to classify patients into risk groups for hospitalization.[2] Since age is considered directly in the score, a continuum of values is possible. It may be noteworthy that assessment of some organ systems relies on anamnestic data only and does not account for current development of disease.

### SCAP

SCAP was proposed by a Spanish group to predict critical time courses of pneumonia.[11] It additionally considers kidney (BUN) and age as prognostic parameters. Due to the weighting of factors, the score is quasi-continuous.

### SIRS-Score

The SIRS-Score according to Reinhart et al. is based on Bone et al.[12]^,^[13] This score is used to distinguish between infection, sepsis or severe sepsis, as well as the systemic inflammatory response syndrome (SIRS). According to Reinhart et al. sepsis was defined as the presence of SIRS in response to an infection.[12] In this sense, we utilize the three SIRS-Score stages (1) infection, (2) sepsis, and (3) severe sepsis to rate CAP severity, i.e. the score has four possible expressions.

### SMART-COP

SMART-COP was proposed to predict requirement of intensive care of pneumonia patients.[14] It was also proposed as a measure of CAP severity. Going beyond the assessment of lung, cardiovascular system, and central nervous system, it also considers the liver (albumin) and metabolism (arterial pH). Due to the weighting of factors the score is quasi-continuous.

### SOFA and qSOFA

The sequential organ failure assessment score (SOFA) was defined to describe the condition of a patient during ICU stay.[15] It was introduced as a six-dimensional scoring system, with sub-scores for each organ system covered: respiratory, cardiovascular, hepatic, coagulation, renal, and neurological. All six sub-scores are categorical with values between 0 and 4 and their evaluation is based on a total of 12 parameters. In order to obtain an overall evaluation, we consider the sum of the six sub-scores as recommended by others.[16–18] The sum-score is quasi-continuous with values between 0 and 24 and is referred to as SOFA score in the following. The qSOFA was proposed as a simplified version of the SOFA score only considering respiratory rate, blood pressure, and mental status, with four different possible values.[19]

Due to the design of the PROGRESS study protocol, CRB-65, CURB, PSI, SIRS, Halm, SCAP, and SMART-COP could only be determined at baseline while SOFA and qSOFA were available throughout study visits.

**Figure S1** Age distribution in the PROGRESS cohort in comparison with AQUA[20]. PROGRESS patients are younger than the overall CAP population described in the AQUA report.


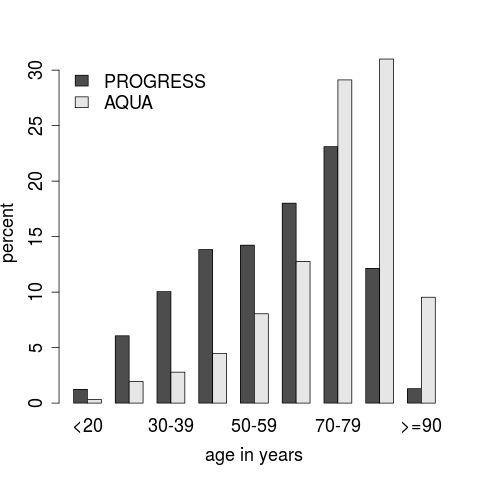


**Figure S2** Distribution of CRB-65 on d0 in comparison with AQUA [20]. PROGRESS patients appear to have less severe disease at enrolment than the overall CAP population described in AQUA. Lower age is partly responsible for this effect.


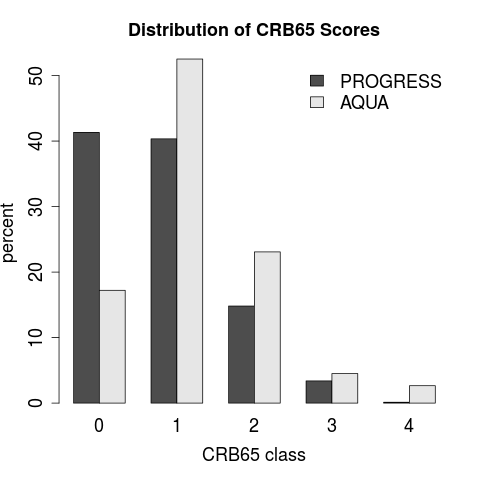


**Figure S3** Distribution of PSI on d0 in comparison with GenIMS[21]. There appears to be a larger fraction of patients with less severe disease and lower mortality risk in PROGRESS compared to patients in GenIMS.


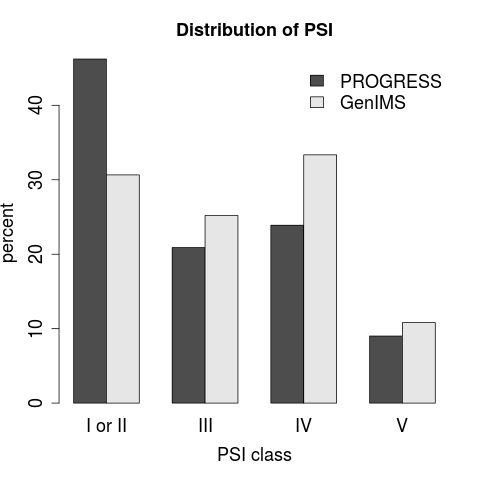


**Figure S4** Distribution of SOFA scores at different time points (d0=enrolment, d1=study visit 1, d2=study visit 2, d3=study visit 3, d4=study visit 4). SOFA scores 7 to 24 were pooled. According to PROGRESS study protocol, patients with initially high disease severity were not subjected to study visits. Therefore, at visit d0 a clear shift towards higher scores was observed. Overall, there appears to be a general trend towards improved SOFA scores over time. However, a few patients still have increased SOFA values at later time points.


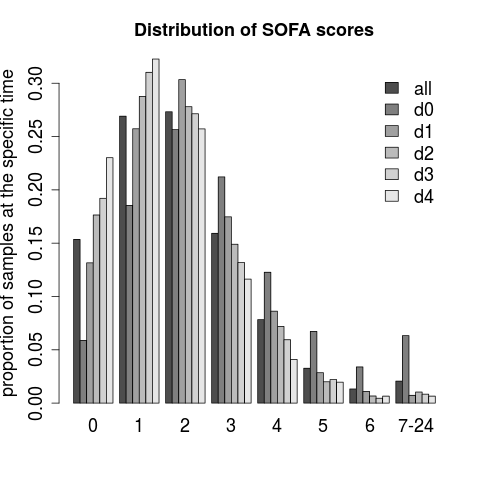


**Table S1:** Net reclassification improvements (NRI) for patients with (cases) and without (controls) PE induced by the scores compared with the null model (guessing) and corresponding NRIs of SOFA compared to the other scores. SOFA is superior to all other scores for both, cases and controls.

|  | Score compared to null model | | SOFA compared to other score | |
| --- | --- | --- | --- | --- |
| score | NRI cases | NRI controls | NRI cases | NRI controls |
| SOFA | 0.73 | 0.76 | - | - |
| ATS | 0.57 | 0.78 | 0.28 | 0.17 |
| SCAP | 0.39 | 0.68 | 0.57 | 0.7 |
| SMART-COP | 0.39 | 0.69 | 0.51 | 0.5 |
| Halm | 0.26 | 0.58 | 0.48 | 0.73 |
| PSI | 0.53 | 0.4 | 0.71 | 0.73 |
| CURB-65 | 0.44 | 0.39 | 0.57 | 0.63 |
| CRB-65 | 0.1 | 0.68 | 0.55 | 0.76 |
| SIRS | -0.1 | 0.91 | 0.53 | 0.65 |
| qSOFA | 0.71 | -0.067 | 0.57 | 0.69 |

References

1. Lim WS. Defining community acquired pneumonia severity on presentation to hospital: an international derivation and validation study. Thorax. 2003;58:377–82. doi:10.1136/thorax.58.5.377.

2. Ebell MH. Outpatient vs. inpatient treatment of community acquired pneumonia. Fam Pract Manag. 2006;13:41–4.

3. Halm EA, Fine MJ, Marrie TJ, Coley CM, Kapoor WN, Obrosky DS, Singer DE. Time to clinical stability in patients hospitalized with community-acquired pneumonia: implications for practice guidelines. JAMA. 1998;279:1452–7.

4. Akram AR, Chalmers JD, Taylor JK, Rutherford J, Singanayagam A, Hill AT. An evaluation of clinical stability criteria to predict hospital course in community-acquired pneumonia. Clin Microbiol Infect. 2013;19:1174–80. doi:10.1111/1469-0691.12173.

5. Mandell LA, Wunderink RG, Anzueto A, Bartlett JG, Campbell GD, Dean NC, et al. Infectious Diseases Society of America/American Thoracic Society consensus guidelines on the management of community-acquired pneumonia in adults. Clin Infect Dis. 2007;44 Suppl 2:S27-72. doi:10.1086/511159.

6. Kolditz M, Ewig S, Klapdor B, Schutte H, Winning J, Rupp J, et al. Community-acquired pneumonia as medical emergency: predictors of early deterioration. Thorax. 2015;70:551–8. doi:10.1136/thoraxjnl-2014-206744.

7. Fine MJ, Auble TE, Yealy DM, Hanusa BH, Weissfeld LA, Singer DE, et al. A prediction rule to identify low-risk patients with community-acquired pneumonia. N. Engl. J. Med. 1997;336:243–50. doi:10.1056/NEJM199701233360402.

8. Fine MJ, Orloff JJ, Arisumi D, Fang GD, Arena VC, Hanusa BH, et al. Prognosis of patients hospitalized with community-acquired pneumonia. The American Journal of Medicine. 1990;88:1N-8N.

9. Fine MJ, Singer DE, Hanusa BH, Lave JR, Kapoor WN. Validation of a pneumonia prognostic index using the MedisGroups Comparative Hospital Database. The American Journal of Medicine. 1993;94:153–9.

10. Hutyrová B, Jakubec P, Šindelářová Z, Šubová J, Langová K, Kolek V. Význam Pneumonia Severity Index u pacientů s pneumonií hospitalizovaných na jednotce intenzivní pneumologické péče. Vnitr Lek. 2015;61:15–23.

11. Espana PP, Capelastegui A, Gorordo I, Esteban C, Oribe M, Ortega M, et al. Development and validation of a clinical prediction rule for severe community-acquired pneumonia. Am J Respir Crit Care Med. 2006;174:1249–56. doi:10.1164/rccm.200602-177OC.

12. Reinhart K, Brunkhorst FM, Bone H-G, Bardutzky J, Dempfle C-E, Forst H, et al. Prävention, Diagnose, Therapie und Nachsorge der Sepsis. 2010. http://www.sepsis-gesellschaft.de/cgi-bin/WebObjects/DsgCMS.woa/3/wr?wodata=Media%2FDSG%2FMedien%2FPDFs%2FSepsis-Leitlinie-2010Leitlinie-Sepsis-2010-05-05.pdf398375550x766. Accessed 18 Apr 2016.

13. Bone RC, Balk RA, Cerra FB, Dellinger RP, Fein AM, Knaus WA, et al. Definitions for sepsis and organ failure and guidelines for the use of innovative therapies in sepsis. The ACCP/SCCM Consensus Conference Committee. American College of Chest Physicians/Society of Critical Care Medicine. Chest. 1992;101:1644–55.

14. Charles PGP, Wolfe R, Whitby M, Fine MJ, Fuller AJ, Stirling R, et al. SMART-COP: a tool for predicting the need for intensive respiratory or vasopressor support in community-acquired pneumonia. Clin Infect Dis. 2008;47:375–84. doi:10.1086/589754.

15. Vincent JL, Moreno R, Takala J, Willatts S, Mendonça A de, Bruining H, et al. The SOFA (Sepsis-related Organ Failure Assessment) score to describe organ dysfunction/failure. On behalf of the Working Group on Sepsis-Related Problems of the European Society of Intensive Care Medicine. Intensive Care Med. 1996;22:707–10.

16. Arts, D G T, de Keizer, N F, Vroom MB, Jonge E de. Reliability and accuracy of Sequential Organ Failure Assessment (SOFA) scoring. Crit. Care Med. 2005;33:1988–93.

17. Ferreira FL. Serial Evaluation of the SOFA Score to Predict Outcome in Critically Ill Patients. JAMA. 2001;286:1754. doi:10.1001/jama.286.14.1754.

18. Minne L, Abu-Hanna A, Jonge E de. Evaluation of SOFA-based models for predicting mortality in the ICU: A systematic review. Crit Care. 2008;12:R161. doi:10.1186/cc7160.

19. Singer M, Deutschman CS, Seymour CW, Shankar-Hari M, Annane D, Bauer M, et al. The Third International Consensus Definitions for Sepsis and Septic Shock (Sepsis-3). JAMA. 2016;315:801–10. doi:10.1001/jama.2016.0287.

20. AQUA - Institut für angewandte Qualitätsförderung und Forschung im Gesundheitswesen GmbH. PNEU – PNEU - Ambulant erworbene Pneumonie. 2015. https://www.sqg.de/downloads/Bundesauswertungen/2014/bu_Gesamt_PNEU_2014.pdf.

21. Kellum JA, Kong L, Fink MP, Weissfeld LA, Yealy DM, Pinsky MR, et al. Understanding the inflammatory cytokine response in pneumonia and sepsis: results of the Genetic and Inflammatory Markers of Sepsis (GenIMS) Study. Arch. Intern. Med. 2007;167:1655–63. doi:10.1001/archinte.167.15.1655.
